# Predictors of teriparatide treatment failure in patients with low bone mass

**DOI:** 10.1016/j.bonr.2015.11.001

**Published:** 2015-11-17

**Authors:** Tarig Elraiyah, Adil H. Ahmed, Zhen Wang, Joshua N. Farr, Mohammad H. Murad, Matthew T. Drake

**Affiliations:** a Department of Molecular Pharmacology and Experimental Therapeutics, Mayo Clinic, Rochester, MN, United States; b Knowledge and Evaluation Research Unit, Mayo Clinic, Rochester, MN, United States; c Wichita Falls Family Practice Residency Program (WFFRP), North Central Texas Medical Foundation, Wichita Falls, TX, United States; d Center for the Science of Healthcare Delivery, Mayo Clinic, Rochester, MN, United States; e Division of Endocrinology, Diabetes, Metabolism, and Nutrition, Mayo Clinic, Rochester, MN, United States; f Division of Preventive, Occupational and Aerospace Medicine, Mayo Clinic, Rochester, MN, United States

**Keywords:** Anabolic skeletal therapy, Osteoporosis, Response failure, Osteoporotic fracture, Bone mineral density

## Abstract

**Introduction:**

While teriparatide is the only skeletal anabolic agent approved in the United States, treatment failure is a major concern which complicates its clinical utility. We sought to identify factors that predict response failure in patients with low bone mass.

**Method:**

We performed a retrospective study of adults with osteopenia or osteoporosis (T-scores < − 1.0 and − 2.5 SD below normal, respectively, at the total hip or lumbar spine) treated with teriparatide at the Mayo Clinic (Rochester, Minnesota) between November 2002–December 2012. Trained study investigators blinded to patient outcomes collected electronic medical record data. Potential response failure predictors were identified using univariate analysis. Multivariable logistic regression modeling was used to identify independent predictors of treatment failure based on either osteoporotic fragility fracture or BMD response.

**Results:**

During the 10-year period, 494 patients received teriparatide treatment and met eligibility criteria. Thirty-five patients had osteoporotic fractures, while 172 did not achieve a ≥ 3% BMD increase. Among predictors as defined by BMD change, both prior bisphosphonate treatment [odds ratio (95% confidence interval), 1.50 (1.01–2.24)] and vitamin D therapy [1.50 (1.01–2.22)] were significantly (P < 0.05) associated with teriparatide treatment failure. By contrast, no predictors were associated with treatment failure when fracture was the endpoint.

**Conclusion:**

These data suggest that prior bisphosphonate or vitamin D exposure may predict response failure to teriparatide therapy. Although these findings may, in part, reflect increased severity or longer duration of disease, this knowledge should help guide clinicians and patients when therapy choices are made.

## Highlights

• A retrospective study of 494 patients was performed to assess the predictors of teriparatide therapy failure.
• Prior treatment with bisphosphonate and vitamin D was found to be independent predictors.
• Future prospective studies are needed to evaluate other factors that are difficult to be assessed retrospectively.

## 1 Introduction

Osteoporosis affects more than 20 million Americans and is associated with approximately 1.5 million fractures annually (Finkelstein et al., 2003). Thus, osteoporosis represents a major health problem that will only worsen as the population ages. The osteoporotic skeleton is characterized by diminished bone mineral density (BMD), reduced bone quality, and increased fragility, all of which increase susceptibility to fractures (Anon., 1993).

Teriparatide [recombinant human parathyroid hormone (PTH)] is a recombinant molecule composed of the amino-terminal 34 amino acids of human PTH (Body et al., 2002). FDA-approved in November 2002, teriparatide is the only currently available skeletal anabolic agent in the United States, and is most frequently reserved for patients with severe osteoporosis or in whom other treatment modalities have failed (Ragucci and Shrader, 2011, Andrews et al., 2012). Daily subcutaneous injection of teriparatide both stimulates osteoblast generation and limits osteoblast elimination by apoptosis (Body et al., 2002), ultimately resulting in new bone formation with increases in both bone mass and strength (Yu et al., 2011). A randomized controlled trial (RCT) conducted by Neer et al. (2001) in 2001 reported that teriparatide significantly increased BMD and reduced both vertebral and non-vertebral fractures when administered (once daily) subcutaneously at doses of either 20 or 40 micrograms (μg).

A potential drawback associated with teriparatide use is response failure. Gallagher et al. (2006) reported this problem in a review of three RCTs that included postmenopausal women treated with teriparatide, in comparison to either placebo or treatment with the bisphosphonate, alendronate. Interestingly, when administered subcutaneously at a daily dose of either 20 or 40 μg, the response rate to teriparatide, as defined by a minimum increase in lumbar spine BMD from baseline of 3%, ranged from 87 to 94% (Gallagher et al., 2006). Nevertheless, the authors were unable to detect any differences in baseline characteristics between patients who responded versus those who did not. In a more recent study, Heaney & Watson (2011) reported that the response rate to teriparatide may be more variable and somewhat lower, with positive BMD response rates of 44.8% and 82.5% at the lumbar spine and total hip, respectively.

Considering that teriparatide treatment is associated with significant cost and treatment burden that includes daily injections, it is of upmost importance to identify predictors of treatment failure. Therefore, we aimed to establish the impact of an array of baseline characteristics and osteoporosis-related exposures on the response to teriparatide treatment in patients with low BMD, and to identify factors that significantly predict treatment failure.

## 2 Material and methods

### 2.1 Study participants and setting

This study was a retrospective analysis carried out using the unique electronic medical record system at the Mayo Clinic, a tertiary care teaching institution located in Rochester, Minnesota. The study protocol was approved by the Mayo Clinic Institutional Review Board and all patients provided authorization for review of their medical records for research in accordance with Minnesota privacy law (St Sauver et al., 2012). To be eligible for study inclusion, patients were required to have received teriparatide (for at least 12 months) at Mayo Clinic between 1 November 2002 and 31 December 2012. We included patients > 18 years old who were diagnosed with low bone mass (osteopenia or osteoporosis with T-scores less than 1.0 and 2.5 SD below normal respectively, at either the total hip or lumbar spine). Medical records with missing outcome data were excluded from analysis. Patients were not otherwise excluded based on specific baseline medical conditions or medications. Outcomes evaluated were the occurrence of osteoporotic fracture and BMD treatment failure (defined as a less than 3% increase from baseline at either the total hip or spine).

### 2.2 Data source

All data were retrieved from the Mayo Clinic Life Sciences System (MCLSS), an exhaustive clinical data warehouse which stores patient demographics, diagnoses, clinical notes, and hospital, laboratory, flow sheet, and pathology data gathered from various clinical and hospital source systems within the institution (Alsara et al., 2011). To conduct the search in MCLSS, we used the query-building tool provided by MCLSS, Data Discovery and Query Builder (DDQB), which allows a thorough interrogation of MCLSS for the intended data (Alsara et al., 2011). To ensure the reliability of this tool, we manually retrieved fracture data from medical records of 20 randomly selected patients. Inter-rater agreement (*k*) between the tool and manual extraction was excellent (*k* = 0.95).

### 2.3 Definitions of treatment failure

Clinical response failure was defined as sustaining one or more osteoporotic fractures (i.e., hip, spine, distal forearm, proximal humerus) after the patient has been treated with teriparatide for at least 6 months. Osteoporotic fractures, also known as fragility or minimal-trauma fractures, were defined by convention as occurring from low-energy trauma such as a fall from a standing height or less, and due to no more than moderate trauma (e.g., motor vehicle accidents) (Rebolledo et al., 2011). Radiographic response failure was defined as < 3% increase in BMD from baseline at the spine, total hip or both when measured at least 12 months following teriparatide initiation (Gallagher et al., 2006). BMD measurements were obtained at time of teriparatide initiation. For study inclusion, subjects must have had a repeat BMD determination performed 12–24 months following treatment initiation. To adjust for this variation in follow-up length from time of treatment initiation, we calculated the average between-measurement time (referred to as follow-up duration) and included this variable in the regression model.

### 2.4 Ascertainment of study variables

Outcome variables were collected by study investigators who retrieved information about fracture occurrence as well as baseline and follow-up BMD measurements from the electronic medical record of the Mayo Clinic. BMD was assessed by dual-energy X-ray absorptiometry (DXA) at the total lumbar spine, total hip and femoral neck using a Lunar Prodigy scanner (General Electric Healthcare, Waukesha, WI), as described previously (Dy et al., 2012). To reduce measurement errors, standard practice at Mayo Clinic is to report the average of 2 scans performed during each assessment. In addition, the average least significant change (LSC) in BMD for all technicians is included in order to avoid the need for patients to have scans performed on the same machine by the same technician each time an assessment is performed. The LSC is defined as the smallest amount of change between two BMD measurements over time that must be exceeded before a change can be considered the result of a true difference in a patient's BMD and not due to either DXA or patient factors with 95% confidence (Shepherd & Lu, 2007).

Predictor variables were identified a priori, and were collected from the electronic medical records using DDQB. Based on opinions received from a panel of content experts, in addition to a comprehensive literature search (i.e. previous studies, meta-analyses and review articles written by experts in the bone and osteoporosis fields), we were able to identify several variables that could be potential predictors of response failure. These factors included: 1) demographics: age and sex; 2) anthropometric measurements: height, weight and body mass index (BMI); 3) habitual exposures: alcohol intake and cigarette smoking; 4) co-morbidities: hypertension, diabetes, cancer, chronic renal disease, chronic liver disease; 5) baseline medications and supplements (i.e. bisphosphonates, corticosteroids, calcium, phosphate, proton pump inhibitors, vitamin D); and 5) biochemical parameters (i.e. bone alkaline phosphatase, C-terminal telopeptide of type I collagen (CTX), 25-hydroxyvitamin D, parathyroid hormone) at baseline and after treatment completion.

### 2.5 Statistical analysis

Data for the predictor variables are presented as means and standard deviations (SD) for variables with normal distribution, and medians and interquartile ranges (IQR) for those with skewed distributions, as appropriate. Categorical variables are presented as frequencies and percentages. Unpaired Student's t-tests were used to compare continuous variables with normal distribution, and the Mann–Whitney U test otherwise. For comparison of categorical variables, chi-square tests were used.

Candidate predictors of response were identified using univariate logistic regression. Predictors that achieved a level of significance equal to a *P*-value of < 0.05 were selected for inclusion in the multivariate model to predict treatment failure (at least one fracture versus no fractures; or less than a 3% increase in total hip and or spine BMD from baseline versus 3% or more). To determine the independent impact of each variable on the response to teriparatide treatment, multivariable logistic regression models were created. We included risk factors identified from univariate models as covariates and either fracture or BMD response failure as dependent variables. For all analyses, a P-value < 0.05 was considered statistically significant. Analyses were performed using STATA, version 12.1 (StataCorp LP, College Station, TX, USA).

## 3 Results

### 3.1 Participants

We included a total of 494 patients who received teriparatide in Mayo Clinic and met the eligibility criteria for the study. Baseline characteristics of the participants, according to fracture and BMD response, are presented in Table 1. All groups were similar in age, sex, race and BMI. Further, the fracture and non-fracture groups were similar at baseline except for the tendency for a higher prevalence of diabetes and hypertension, and prior bisphosphonate treatment in the fracture group. In addition, when compared to those who achieved the a priori chosen BMD response, the group that did not achieve this response contained more current smokers and included a greater proportion of patients who had previously been treated with bisphosphonate therapy.

Although we were unable to measure adherence with the prescribed treatment directly, high compliance in this population is assumed given the rigorous methods followed by the Mayo Clinic specialty pharmacy in assuring timely prescription refills and medication delivery, a process which includes monthly phone call interaction with each patient prior to medication issuance. In addition, we excluded all patients who did not have both pre- and post-treatment BMD determinations as defined in the methods section.

### 3.2 Predictors of treatment failure based on fracture incidence

In univariate analyses, multiple factors were significantly more common in the non-response group when defined by fracture incidence. These included age, BMI, sex, baseline comorbidities: hypertension and malignancy, and baseline medications: bisphosphonate, calcium, corticosteroid, proton pump inhibitors (PPIs) and vitamin D. In comparison, multivariable modeling to predict non-response as defined by the occurrence of at least one osteoporotic fracture did not identify any of these as a significant predictor. Table 2 shows the multivariate-adjusted analyses based on fracture incidence.

### 3.3 Predictors of treatment failure based on BMD change

Univariate analyses yielded multiple variables which were significantly more common in the group that failed to respond by BMD difference from baseline, including age, sex, baseline comorbidities: hypertension and malignancy, and baseline medications: bisphosphonate, calcium, corticosteroid, PPIs and vitamin D. When adjustment for these variables using multivariate logistic regression models was performed, however, only two significant baseline predictors of treatment failure were identified: bisphosphonate therapy (OR = 1.50; 95% CI: 1.01–2.24; P = 0.045) and vitamin D treatment (OR = 1.50; 95% CI: 1.01–2.22; P = 0.043). Table 3 presents the multivariate-adjusted analyses based on BMD failure.

To compare the effect of different types of bisphosphonate on response failure, we measured the OR of non-response for the group that received alendronate or zoledronic acid compared to the group that received other types of bisphosphonate. No statistically significant difference in response failure was detected between the 2 groups (OR 0.70; 95% CI: 0.26–1.90; P = 0.49).

## 4 Discussion

We conducted a retrospective cohort study to identify possible predictors of treatment failure with teriparatide. Although approved as the only skeletal anabolic agent in the United States, treatment with teriparatide is associated with significant cost, as well as a significant burden on patients that includes daily injections. We found that after inclusion of several important potential factors that may predict response failure to teriparatide, only prior bisphosphonate treatment and vitamin D therapy were significant predictors.

One possible explanation for these findings may be that patients who have received such prior treatment have more severe osteoporosis or disease of longer duration. An alternative (and not mutually exclusive explanation) is that prior bisphosphonate therapy has led to suppression of bone turnover, ultimately leading to an impaired response to teriparatide, as teriparatide action requires both increased osteoblast and osteoclast activity — with osteoblast (bone building) activity predominating.

With regard to the demonstrated effect of vitamin D supplementation, a plausible explanation is that patients receiving vitamin D supplementation may be at increased risk of underlying osteomalacia or incompletely healed osteomalacia, and that vitamin D has been provided to assist with healing. Accordingly, this baseline skeletal hypomineralization may have impaired the BMD response to teriparatide treatment. An alternative explanation is that this effect could be related to the role of vitamin D as an inducer of osteoclast formation via its promotion of receptor activator of nuclear factor-kB ligand (RANKL) expression. This effect of vitamin D supplementation has been hypothesized to increase bone resorption and decrease the efficacy of bisphosphonates in patients with metastatic bone tumors (Altundag et al., 2004). A recent study showed that this effect can be reversed in vivo by demonstrating that daily administration of active vitamin D compounds suppresses bone resorption in animal models (Takahashi et al., 2014). Nevertheless, the in vitro negative effect of vitamin D could still explain its association with a decreased skeletal anabolic response when combined with teriparatide, particularly given that teriparatide itself increases osteoclast activity.

To our knowledge, our study is the first to attempt to identify predictors of treatment failure in subjects treated with teriparatide. A recent study by Diez-Perez et al. (2014) assessed predictors for anti-osteoporosis medications in general. While their analysis included prospectively collected data and therefore allowed assessment of some variables for which we were unable to test, our study design allowed us to collect data on vitamin D supplementation and BMD, factors which to our knowledge have not been previously studied. Of note, our findings with regards to bisphosphonate treatment are supported by two studies that evaluated the effect of bisphosphonate therapy on BMD change (Ettinger et al., 2004, Koski et al., 2013), both of which demonstrated that prior bisphosphonate treatment was associated with reduced BMD response. In contrast, however, a retrospective study from the UK reported that no differences were detected in BMD change from baseline when patients were stratified based on whether or not they had received prior bisphosphonate therapy (Middleton et al., 2007), results similar to those reported in another European study (Boonen et al., 2008). In comparison, a study that evaluated teriparatide treatment following treatment with either alendronate or risedronate found that increases in BMD above 3% at the lumbar spine did still occur following bisphosphonate pretreatment but were greater in patients who had previously been treated with risedronate, suggesting that the skeletal anabolic response to teriparatide differs based on prior bisphosphonate therapy (Miller et al., 2008).

Our study has several limitations inherent to the design. For example, it is prone to potential bias due to missing data because some of the variables studied were, in limited cases, not adequately documented in the medical record. We acknowledge this may have affected our final analyses, although given the relatively few instances of missing data, this issue likely had limited impact on our findings. It should also be noted that such studies are inherently susceptible to exposure ascertainment bias. To prevent this, investigators were blinded to the outcome status of the patient, and we ensured that exposure ascertainment was done similarly for all subjects. Another threat to our results is under reporting of fracture occurrence, particularly since vertebral fractures are frequently subclinical and thus not always diagnosed (Gehlbach et al., 2000, Delmas et al., 2005, Hajcsar et al., 2000, Kim et al., 2004).

Despite these limitations, our study has significant strengths, including our ability to specifically study the predictors of teriparatide failure using data identified directly from real clinical practice. Importantly, such data are direly needed yet currently very scarce; thus, our study has considerable merit. Indeed, our study included the largest longitudinally followed (spanning the course of an entire decade) cohort of patients treated with teriparatide to date, and also included both men and women with low bone mass without restriction to specific age group, thereby providing broader applicability to its inferences.

Collectively, the baseline variables tested in our study were unable to fully predict teriparatide treatment failure in low bone mass patients. Given the limitations associated with our study design, prospective observational studies that allow for better control over study variables will likely provide more conclusive results, as such designs allow for the collection of additional factors that we were unable to assess (e.g., falls, physical activity and function, and dietary intakes). It is also worth mentioning that with only 35 fracture patients, our study may have been underpowered to detect significant predictors of this endpoint. Therefore, future larger studies to address this particular endpoint are needed.

In terms of the practical implications of our findings, the data presented here are useful for informing patients and physicians contemplating treatment decisions. Although teriparatide is currently the only available skeletal anabolic agent, optimal shared decision making requires conveying the uncertainty to patients. Informing patients about the possibility of - and potential predictors for - treatment failure, and providing them with quantitative estimates about an expensive treatment that requires daily injection is important, and will help patients to make decisions consistent with their values and preferences.

## 5 Conclusion

Prior treatment with bisphosphonate and vitamin D are independent predictors of teriparatide therapy failure. Further prospective studies are needed to establish whether these findings reflect increased severity or longer duration of disease, as well as additional factors that predict treatment failure response to teriparatide. Such knowledge should help patients and clinicians engage in shared decision making when therapy choices are made.

## Conflict of interest

Tarig Elraiyah, Adil H. Ahmed, Zhen Wang, Joshua N. Farr, Mohammad H. Murad, and Matthew T. Drake declare that they have no conflict of interest.

## Financial support

The authors received no financial support to conduct this study.

## Figures and Tables

**Table 1:** Baseline characteristics by BMD response and fracture group.

	Fracture	No fracture	*P*	BMD failure	BMD response	*P*
N (%)	35 (7.1)	459 (92.9)	…	172 (34.8)	322 (65.2)	…
Sex			0.494			0.323
Male	5 (14.3)	87 (18.9)		28 (16.3)	64 (19.8)	
Female	30 (85.7)	372 (81.1)		144 (83.7)	258 (80.2)
Age	62.4 (12.4)	66.2 (12.6)	0.890	65.7 (12.2)	66.0 (12.8)	0.862
Race			0.662			0.586
Caucasian	35 (100)	439 (95.6)		163 (94.8)	311 (96.6)
African American	0 (0)	3 (0.7)		1 (0.6)	2 (0.6)
Asian	0 (0)	6 (1.3)		2 (1.2)	4 (1.2)
Other	0 (0)	11 (2.4)		6 (3.4)	5 (1.6)
Follow up (months) ^a^	21 (17, 25)	14 (12, 22)	0.001	14 (12, 23)	15 (13, 22)	0.707
BMI (kg/m^2^)	25.2 (5.9)	25.0 (5.1)	0.850	24.8 (5.2)	25.1 (5.1)	0.620
Weight (Kg)	62.7 (21.9)	66.0 (16.4)	0.285	64.5 (17.5)	66.4 (16.5)	0.247
Smoking status			0.240			0.036
Current	3 (8.6)	20 (4.3)		13 (7.5)	10 (3.1)	
Past	6 (17.1)	118 (25.7)		36 (20.9)	88 (27.3)
Never	19 (54.3)	190 (41.4)		69 (40.1)	140 (43.5)
Unknown	7 (20)	131 (28.5)		54 (31.4)	84 (26.0)
Comorbid conditions						
Asthma	4 (11.4)	25 (54.5)	0.147	11 (6.4)	18 (5.6)	0.716
COPD	3 (8.6)	18 (3.9)	0.189	7 (4.1)	14 (4.3)	0.884
CAD	2 (5.7)	30 (6.5)	0.849	10 (5.8)	22 (6.8)	0.661
Diabetes	6 (17.1)	33 (7.2)	0.035	11 (6.4)	28 (8.7)	0.366
Hypertension	13 (37.1)	100 (21.8)	0.037	41 (23.8)	72 (22.4)	0.710
Liver disease	0 (0)	2 (0.4)	1.000	0 (0)	2 (0.6)	1.000
Malignancy	5 (14.3)	67 (14.6)	0.960	24 (13.9)	48 (14.9)	0.775
Renal disease	2 (5.7)	11 (2.4)	0.237	5 (2.9)	8 (2.5)	0.780
Baseline medication use						
Bisphosphonates^b^	18 (51.4)	158 (34.4)	0.043	74 (43.0)	102 (31.7)	0.012
Corticosteroids	12 (34.3)	95 (20.7)	0.060	41 (23.8)	66 (20.5)	0.391
Calcium	13 (37.1)	109 (23.7)	0.076	47 (27.3)	125 (38.8)	0.322
Phosphate	0 (0)	3 (6.5)	1.000	0 (0)	3 (0.9)	1.000
PPIs	11 (31.4)	85 (18.5)	0.063	32 (18.6)	64 (19.9)	0.734
Vitamin D	23 (65.7)	227 (49.4)	0.064	100 (58.1)	150 (46.6)	0.014
Baseline lab values^a^^,^^c^						
BAP (μg/L)	18 (10.45, 24)	14 (10.5, 22)	0.725	12 (10, 22)	16 (11, 21.7)	0.542
CTX (pg/mL)	167 (139, 1188)	668 (396, 899)	0.282	515 (352, 1235)	668 (288, 811)	0.650
Vitamin D (ng/mL)	33 (27, 51)	35 (29.5, 47)	0.694	36 (29.5, 49.5)	35 (28, 45)	0.638
PTH (pg/mL)	37 (23, 49)	27 (22, 37.5)	0.476	26 (19.5, 37)	30 (23, 41)	0.409

Data are presented as mean (SD) or number (percentage), unless specified otherwise.

a Data are presented as median (Q1, Q3).

b The types of bisphosphonate used by patients in this study included: alendronate, etidronate, ibandronate, risedronate, zoledronic acid.

c Reference ranges are as follows: BAP: adult male < 20 μg/L, adult premenopausal female < 14 μg/L, postmenopausal female < 22 μg/L; CTX: adult male 35–836 pg/mL, adult premenopausal female 25–573 pg/mL, adult postmenopausal female 104–1008 pg/mL; Vitamin D 20–50 ng/mL; PTH 15–65 pg/mL.

**Table 2:** Multivariate-adjusted analyses based on fracture incidence.

	Fracture (n = 35)	No fracture (n = 459)	OR (95% CI)	*P* value
Baseline demographics				
Age, mean (SD)	62.4 (12.4)	66.2 (12.6)	0.98 (0.94–1.01)	0.109
BMI, mean (SD)	25.2 (5.9)	25.0 (5.1)	1.01 (0.94–1.09)	0.737
Gender, male/female (%)	5/30 (14.3/85.7)	87/372 (18.9/81.1)	0.76 (0.26–2.24)	0.621
Baseline co-morbidities				
Hypertension, n (%)	13 (37.1)	100 (21.8)	2.05 (0.91–4.63)	0.085
Malignancy, n (%)	5 (14.3)	67 (14.6)	1.05 (0.37–2.97)	0.931
Baseline medications				
Bisphosphonate, n (%)	18 (51.4)	158 (34.4)	1.25 (0.57–2.76)	0.580
Calcium, n (%)	13 (37.1)	109 (23.7)	1.73 (0.79–3.82)	0.171
Corticosteroids, n (%)	12 (34.3)	95 (20.7)	1.33 (0.58–3.02)	0.502
PPIs, n (%)	11 (31.4)	85 (18.5)	1.67 (0.73–3.83)	0.229
Vitamin D, n (%)	23 (65.7)	227 (49.4)	1.53 (0.68–3.43)	0.307

Abbreviations: SD: standard deviation; OR: odds ratio; BMI: body mass index; PPIs: proton pump inhibitors.

**Table 3:** Multivariate-adjusted analyses based on BMD failure:

	BMD failure (n = 172)	BMD response (n = 322)	OR (95% CI)	*P* value
Baseline demographics				
Age, mean (SD)	65.7 (12.2)	66.0 (12.8)	1.00 (0.98–1.01)	0.890
Gender, male/female (%)	28/144 (16.3/83.7)	64/258 (19.8/80.2)	0.89 (0.53–1.48)	0.649
Baseline co-morbidities				
Hypertension, n (%)	41 (23.8)	72 (22.4)	1.06 (0.67–1.69)	0.795
Malignancy, n (%)	24 (13.9)	48 (14.9)	0.94 (0.54–1.61)	0.816
Baseline medications				
Bisphosphonate, n (%)	74 (43.0)	102 (31.7)	1.50 (1.01–2.24)	0.045^a^
Calcium, n (%)	47 (27.3)	125 (38.8)	1.19 (0.77–1.84)	0.434
Corticosteroids, n (%)	41 (23.8)	66 (20.5)	1.08 (0.68–1.73)	0.744
PPIs, n (%)	32 (18.6)	64 (19.9)	0.84 (0.51–1.39)	0.509
Vitamin D, n (%)	100 (58.1)	150 (46.6)	1.50 (1.01–2.22)	0.043^a^

Abbreviations: BMD: bone mineral density; SD: standard deviation; OR: odds ratio; PPIs: proton pump inhibitors.

a Independent significant predictors.
